# Overcoming high level adenosine-mediated immunosuppression by DZD2269, a potent and selective A2aR antagonist

**DOI:** 10.1186/s13046-022-02511-1

**Published:** 2022-10-14

**Authors:** Yu Bai, Xin Zhang, Jie Zheng, Ziyi Liu, Zhenfan Yang, Xiaolin Zhang

**Affiliations:** 1 Biomed-X Center, Academy for Advanced Interdisciplinary Studies, Peking University, 100871 Beijing, China; 2 Dizal Pharmaceuticals, 199 Liangjing Rd, Zhangjiang Hi-Tech Park, Pudong District, 201203 Shanghai, China

**Keywords:** DZD2269, Adenosine, A2aR, Immunosuppression

## Abstract

**Background:**

Adenosine is a potent immunosuppressant whose levels in the tumor microenvironment (TME) are often much higher than those in normal tissues. Binding of adenosine to its receptor A2aR activates a cascade of genes and leads to immunosuppression. In addition, immune checkpoint blockage markedly increases A2aR expression in T cells, which could dampen their anti-tumor response. Several A2aR antagonists are under clinical development, but with limited clinical benefit reported so far. These A2aR antagonists showed much diminished activity at high adenosine levels found in TME, which may explain their clinical underperformance. We report the discovery and early clinical development of DZD2269, a novel A2aR antagonist which can fully block A2aR mediated immunosuppression commonly found in TME. Adenosine stimulates phosphorylation of cyclic AMP response element binding protein (CREB) in T cells and inhibits anti-tumor cytokine secretion in PBMCs in a dose-dependent manner. DZD2269 was able to reverse the immunosuppression induced by high concentrations of adenosine, as demonstrated by inhibiting CREB phosphorylation in T cells, restoring Th1 cytokine secretion in PBMCs, and stimulating dendritic cells (DCs) maturation. As a single agent, DZD2269 showed anti-tumor growth in multiple syngeneic mouse tumor models, and more profound anti-tumor effects were observed when DZD2269 was in combination with immune checkpoint inhibitors, radiotherapy, or chemotherapy. A good PK/PD relationship was observed in these animal models. In the phase 1 clinical study, downregulation of pCREB was detected in human T cells, consistent with preclinical prediction. Our data support further clinical development of DZD2269 in patients with cancer.

**Methods:**

The selectivity of DZD2269 for adenosine receptors was tested in engineered cell lines, and its efficacy in blocking A2aR signaling and reversing adenosine-mediated immunosuppression was assessed in human T cells and peripheral blood mononuclear cells (PBMCs). The anti-tumor effects of DZD2269 were evaluated in multiple syngeneic mouse models as a single agent as well as in combination with chemotherapy, radiotherapy, or immune checkpoint inhibitors. A phase 1 study in healthy volunteers (NCT04932005) has been initiated to assess safety, pharmacokinetics (PK) and pharmacodynamics (PD) of DZD2269.

**Results:**

Adenosine stimulates phosphorylation of cyclic AMP response element binding protein (CREB) in T cells and inhibits anti-tumor cytokine secretion in PBMCs in a dose-dependent manner. DZD2269 was able to reverse the immunosuppression induced by high concentrations of adenosine, as demonstrated by inhibiting CREB phosphorylation in T cells, restoring Th1 cytokine secretion in PBMCs, and stimulating dendritic cells (DCs) maturation. As a single agent, DZD2269 showed anti-tumor growth in multiple syngeneic mouse tumor models, and more profound anti-tumor effects were observed when DZD2269 was in combination with immune checkpoint inhibitors, radiotherapy, or chemotherapy. A good PK/PD relationship was observed in these animal models. In the phase 1 clinical study, downregulation of pCREB was detected in human T cells, consistent with preclinical prediction.

**Conclusion:**

DZD2269 is a novel A2aR antagonist which can fully block A2aR mediated immunosuppression commonly found in TME. Clinical development of DZD2269 in patients with cancer is warranted (NCT04634344).

**Supplementary information:**

The online version contains supplementary material available at 10.1186/s13046-022-02511-1.

## Background

Extracellular adenosine is a potent immunosuppressive metabolite. While its level is normally low and constant in most tissues, in response to hypoxic stimulation and inflammation, adenosine level could be induced over a hundred folds [1–3]. The main source of extracellular adenosine is extracellular ATP. In tumor microenvironment, where hypoxia and inflammation are common, adenosine concentration is reported to be even higher, due to higher level of cell death, resulting in the release of large amounts of ATP from dying cells to tumor microenvironment (TME) [3–7]. Extracellular ATP is quickly hydrolyzed by CD39 (ecto-nucleoside triphosphate diphosphohydrolase-1, E-NTPDase1) into ADP or AMP, with the AMP produced further hydrolyzed by CD73 (ecto-5’-nucleotidase, Ecto5’NTase) into adenosine to create an anti-inflammatory milieu [8, 9]. CD38/CD203a can also convert NAD + into AMP and further into adenosine by CD73 [10]. In most tissues, CD73 is the main enzyme that converts AMP into adenosine while prostatic acid phosphatase (PAP) and alkaline phosphatase (ALP) can also convert AMP into adenosine in some tissues and under certain pH conditions [11].

Adenosine affects many aspects of immune cells. It could promote the differentiation and proliferation of regulatory T (Treg) cells and myeloid-derived suppressor cells (MDSCs) [12], block natural killer T (NKT) cell activation [13, 14], inhibit chemokines and reactive oxygen species (ROS) production from neutrophils [15], suppress LPS-induced production of TNF-α by monocytes and dendritic cells (DCs), and suppress effector T cell functions [16, 17]. Adenosine exerts its biological effect through four adenosine receptors, A1R, A2aR, A2bR and A3R [18]. Available evidence suggests that A2aR is the key mediator for its immune suppressive effect [13, 19–21]. Adenosine binding to A2aR receptor activates adenylyl cyclase (AC) and raises intracellular cyclic adenosine monophosphate (cAMP) production and subsequently activates the cAMP-dependent protein kinase A (PKA). The activated PKA phosphorylates the cyclic AMP response element binding protein (CREB) at serine 133 (Ser133), through which regulates the expression of a variety of genes [21].

Given the immunosuppressive nature of the adenosine in TME, it is not surprising that blockade of this pathway has attracted lots of attention for immunotherapies, either through inhibiting adenosine production or directly blocking the activation of its receptor A2aR. The finding that PD-1/L1 inhibitors increase A2aR expression in tumor-infiltrating CD8 + T cells further stimulates the interests [22]. Several anti-CD73 antibodies and A2aR antagonists are at early-stage clinical investigation, as a single agent or in combination with anti-PD-1/L1 antibodies. So far, the published data shows that although these A2aR antagonists are active drugs, the overall response rates are low to moderate [23–26]. The preclinical data has shown that these agents are potent A2aR antagonists when measured at low adenosine level found in normal physiological conditions [27–29]. They all, however, lose their potency rapidly as the adenosine concentration increases. At the adenosine level found in TME, these A2aR antagonists could not effectively inhibit the pathway.

DZD2269 was designed as a potent and selective A2aR antagonist, capable of blocking adenosine-mediated immunosuppression at the therapeutically relevant high adenosine concentrations. We previously showed that DZD2269 could enhance cell killing with radiation in rodent models [30]. Here, we reported that DZD2269 could, while others failed to, completely block the A2aR pathway at high adenosine concentrations equivalent to those in TME. DZD2269 alone showed anti-tumor activity in immunocompetent mouse models, and when in combination with radiotherapy, chemotherapy, or anti-PD-1 antibody, its anti-tumor effect was significantly enhanced. Results from the ongoing phase 1 study demonstrated that DZD2269 effectively inhibited the A2aR pathway at doses without any drug related adverse effects in human. Further development of DZD2269 in multiple disease settings is warranted to further evaluate its safety and efficacy as monotherapy as well as in combination with other treatment modalities.

## Materials and methods

### Cell lines

CHO cells stably expressing human adenosine receptors (CHO-A2aR, CHO-A2bR, CHO-A1R and CHO-A3R) were purchased from GenScript. Cell lines RM-1, B16F10 and Pan02 were obtained from the American Type Culture Collection (ATCC). Cryopreserved human PBMCs were purchased from AllCells. All CHO cells were cultured in Ham’s F12K medium (Gibco) with 10% FBS (Gibco) and RM-1 in DMEM (Gibco) medium with 10% FBS. The Pan02, B16F10 and PBMC cells were grown in RPMI 1640 (Gibco) medium containing 10% FBS. PBMCs were cultured in the presence of 1% penicillin-streptomycin. All other cells were cultured without antibiotics. All cells were maintained and propagated in a humidified incubator at 37 °C, 5% CO_2_.

### Compounds and antibodies

NECA, adenosine and LPS were purchased from Sigma-Aldrich. Oxaliplatin, CPI-444, AZD4635 and AB928 were purchased from MedChemExpress, DZD2269 was designed and synthesized by Dizal Pharmaceuticals. All the compounds were dissolved in DMSO to prepare a 10 mM stock solution and stored in a nitrogen cabinet before use. Anti-PD-1 (BE0146) and Isotype control antibodies were purchased from Bioxcell. Anti-mouse CD45 PE (clone 30-F11), anti-mouse CD4 PerCP (clone RM4-5), anti-mouse CD8a FITC (clone 53 − 6.7), anti-CD45 FITC (clone HI30), anti-human CD8 PE (clone RPA-T8), anti-human CD4 FITC (clone PRA-T4), anti-CD14 PE (clone M5E2), anti-HLA-DR APC-H7 (clone G155-178), anti-CD83 APC (clone HB15e), anti-CD4 (clone, RPA-T4) and anti-CD8 (clone RPA-T8) were purchased from BD Bioscience. Anti-pCREB^Ser133^ Alexa Fluor 647 (clone 87G3) and anti-mouse CD8 (clone D4W2Z) were purchased from Cell Signaling Technology. Anti-human CD3 antibody (clone OKT3) and anti-human CD28 antibody (clone CD28.2) were purchased from eBioscience. Anti-mouse CD3 antibody (clone 145-2C11) and anti-mouse CD28 antibody (clone E18) were purchased from BioLegend. Anti-mouse CD4 (EPR19514) was purchased from Abcam.

### cAMP accumulation assay

Assays were performed with HTRF cAMP kit (Cisbio, 62AM4PEJ). A series dilution of compound (11 concentrations of compound, starting at 10 µM, 4-fold dilutions) was added into 384-well plates prior to cell seeding and DMSO was added to the control well. CHO-A2aR and CHO-A2bR cells were seeded in the plates at a density of 5,000 cells/well in F-12 medium containing 10% FBS. Plates were incubated at 37˚C for 30 min, then DMSO (control), adenosine (1, 10, and 100 µM), or NECA (0.1, 1, and 10 µM) were added to cells and further incubated at 37˚C 30 min. cAMP signal was measured on Envision (Perkin Elmer). The concentration of compound producing 50% inhibition of the cAMP (IC_50_) was calculated using four-parameter logistic fit with XLFIT. The dose-response curves were fitted by nonlinear regression using GraphPad Prism.

NECA is a stable analogue of adenosine commonly used to measure adenosine pathway activity. Consistent with the trend in binding affinity to A2aR [31], NECA was also more potent than adenosine in stimulating cAMP accumulation via A2aR, approximately 10-fold stronger (Supplemental Fig. S1).

### Calcium mobilization assay

Assays were performed with FLIPR Calcium 5 Assay kit (Molecular Devices, R8186). CHO-A1R and CHO-A3R cells were seeded in 384-well plate (10,000 cells/well) in F-12 medium containing 10% FBS. After overnight incubation, the supernatant was removed, and 0.5× loading buffer was added into each well and incubate with cells for additional 60 min at 37 °C. A series diluted compound (11 concentrations of compound, starting at 10 µM, 4-fold dilutions) was added and then stimulated cells with adenosine (1, 10, and 100 µM) or NECA (0.1, 1, and 10 µM), calcium signal was measured on FLIPR. DMSO was added as no stimulation control. The concentration for 50% inhibition of the calcium signal (IC_50_) was calculated using four-parameter logistic fit with XLFIT. The dose-response curves were fitted by nonlinear regression using GraphPad Prism.

### PBMC cytokine release assay

PBMCs (2 × 10^5^ cells/well) were seeded in 96 well plate pre-coated with 10 ng/ml anti-CD3 antibody and stimulated with 20 ng/ml anti-CD28 antibodies to test IFN-γ and IL-2 secretion [32]) or stimulated with 100 ng/ml LPS for TNF-α detection [32]. A series diluted DZD2269 and 10 µM NECA were then added. Cell supernatants were collected at 24 h (IL-2 and TNF-α) and 72 h (IFN-γ) post-stimulation. IL-2 (R&D, S2050), IFN-γ (R&D, SIF50) and TNF-α (eBioscience, 88-7346-88) ELISA kits were used for measurement.

For testing the effect of NECA on activated PBMCs, PBMCs (2 × 10^5^ cells/well) were seeded in 96 well plate precoated with 10 ng/ml anti-CD3 antibody and stimulated with 20 ng/ml anti-CD28 antibody, and simultaneously various concentrations of NECA (0.01, 0.1, 1, 10, 100 µM) and DMSO (Control) were added. IFN-γ was detected in cell supernatants 72 h after stimulation.

### Splenocytes collection

Mouse spleens were removed from C57BL/6 mice (6–8 weeks old, SPF) and placed in ice-cold phosphate-buffered saline (PBS, Gibco). Single-cell suspensions were prepared by gently dispersing cells and filtering through a 70-µm nylon filter and washing twice with PBS. Red blood cells were lysed with ACK lysis buffer (Gibco, A10492) and washed twice with PBS. Lymphocytes were resuspended in RPMI 1640 medium (Gibco) containing 10% FBS.

### Mouse splenocytes cytokine release assay

Mouse splenocytes and irradiated mouse RM-1 cells were mixed and seeded in a U-bottom 96-well plate pre-coated with anti-mouse CD3 antibody overnight, and then anti-mouse CD28 antibody was added to each well. Cell supernatants from co-cultured cells were collected at 24 h (IL-2) and 72 h (IFN-γ) post-stimulation. IL-2 (R&D, SM2000) and IFN-γ (R&D, PMIF00) ELISA kits were used for measurement.

### CREB phosphorylation assay

Whole blood samples from three different healthy donors were collected and seeded in 96-well deep plates at a concentration of 90 µL/well. Add various concentrations of NECA (0.1, 1, 10, 100 µM) and incubate for 15 min at 37 °C to stimulate the phosphorylation of CREB. Cells were then lysed and fixed with BD Lyse/Fix Buffer (BD Bioscience, 558,049) for 15 min at 37 °C. After washed twice with DPBS (Hyclone, SH30028.02), the cells were centrifuged at 600 × g for 8 min. Cells were permeabilized by slow addition of BD Phosflow™ Perm Buffer III (BD Bioscience, 55,805) and incubated on ice for 60 min, and then resuspended in DPBS and stained with anti-CD3, anti-CD4, anti-CD8, and anti-pCREB (Ser133) antibodies for 1 h at room temperature in the dark. Cells were then washed twice and acquired on a flow cytometric (BD FACSCanto™ II) with DIVA software (BD Biosciences). The reported pCREB signal is the mean fluorescent intensity (MFI).

### pCREB signaling inhibition assay

For ex vivo pCREB assessment, whole blood samples were collected from healthy donors, plated at 90 µL/well in 96-well deep plates, and pre-incubated with DZD2269 at 37 °C for 30 min. NECA at 10 µM concentration was then added to the blood samples for 15 min at 37 °C. and then following the same procedure as CREB phosphorylation assay to assess changes of pCREB levels.

For pCREB assessment in phase 1 clinical study, whole blood samples were collected from healthy subjects at pre-dose, 2 and 24 h after DZD2269 single dosing. Blood samples were stimulated ex vivo with or without 10 µM NECA for 15 min at 37 °C, and then following the same procedure as described above to assess changes of pCREB levels. Inhibition of pCREB was normalized to the signal from pre-dose samples.

### DC maturation assay

Monocytes were isolated from human PBMCs using untouched human monocytes kit (Invitrogen, #11350D) according to the manufacturer instruction. Isolated monocytes were cultured with 100 ng/ml GM-CSF (R&D, 215-GM) and 50 ng/ml IL-4 (R&D, 204-IL) supplemented every 2 days. On day 5, 100 ng/ml LPS (Sigma, L4391) was added for 48 h to stimulate DC maturation. NECA and A2aR antagonists were added 30 min before LPS stimulation. CD83 was measured as DC maturation marker by flow cytometry, and the gating strategy is shown in supplemental Fig. S2. Data were analyzed with FlowJo software.

### Animals

All animals (4- to 10-week-old specific pathogen-free) were purchased from Vital River Laboratory Animal Technology Co., Ltd. All studies involving animals were conducted according to the guidelines approved by Institutional Animal Care and Use Committees (IACUC) and the standard and local regulatory requirements of Dizal Pharmaceuticals. For animal welfare, mice were euthanized when body weight loss reached ≥ 20%.

### Establishment of subcutaneous model

B16F10, RM-1 and Pan02 cells were harvested and suspended in DPBS. Single-cell suspensions of greater than 90% viability were used for injection. Tumor cells were injected subcutaneously in the left flank of the C57BL/6 mice with a total cell number of 2 × 10^6^ (B16F10 and RM-1) and 1 × 10^7^ cells (Pan02) in a volume of 0.1 ml. Tumor-bearing mice were randomized into treatment groups when the mean tumor volume reached 40 to 100 mm^3^. Mice were then treated with vehicle or compounds starting the day after randomization, and tumor volume and body weight were measured twice a week. Tumor growth inhibition from the onset of treatment was assessed by comparing the mean change in tumor volume between control and treatment groups and expressed as tumor growth inhibition. Tumor nodules were measured two-dimensionally with calipers, and tumor volumes were calculated using the following formula: tumor volume (TV) = (length × width^2^) × 0.52.

### Establishment of lung cancer metastasis model

Cell suspension containing 2.5 × 10^5^ B16F10 cells in 100 µL PBS was injected into C57BL/6 mice or BALB/c nude mice through tail vein. Mice were administered with DZD2269 1 h before cell injection, twice a day via oral gavage for 13 days. Efficacy was measured by counting tumor modules in the lung after 13-day treatment.

### Immunohistochemistry (IHC) assay

Tumor tissues were collected at the end of the study. The tissues were fixed in 10% neutral buffered formalin overnight following standard procedures for processing, paraffin-embedding, and sectioning for IHC assay [33]. The sections were incubated with primary antibodies for CD8 or CD4 for 1 h at room temperature, and then with Envision + System HRP Labelled Polymer Anti-Rabbit (DAKO, K4003) for 30 min and developed in diaminobenzidine substrate for 5 min. After that, the sections were counterstained, dehydrated and cleared in the Leica XL autostainer. Positive cell percentage of stained IHC slides was quantified with a HALO image analysis platform (Indica Labs).

### Phase 1 clinical study (ClinicalTrials.gov Identifier: NCT04932005)

This is a phase 1, randomized, double-blinded, placebo-controlled study in healthy volunteers. The objectives of this study are to assess safety, tolerability, and pharmacokinetics of DZD2269 as oral tablet. This article reported the PK/PD correlation analysis in single ascending dose cohorts. In these cohorts, healthy subjects were randomly assigned (3:1) to receive DZD2269 (n = 6) or matching placebo (n = 2) in each cohort. The doses of DZD2269 ranged from 5 mg to 160 mg under fasting condition. Whole blood samples were collected from healthy subjects at pre-dose, 2 h, and 24 h after a single dose of DZD2269 for pCREB measurement. Plasma samples from multiple time points, including those for PD assessment, were also collected for PK analysis. Adverse events were monitored and collected throughout the study, from informed consent until the safety follow-up visit.

### Statistical analysis

The comparison of tumor volume in different treatment groups was performed by using two-way ANOVA. The comparison of metastatic nodes numbers, cytokine secretion and T cell infiltration were performed by using one-way ANOVA. All analyses were performed using GraphPad Prism software. P values smaller than 0.05 were considered statistically significant.

## Results

### Adenosine stimulates CREB phosphorylation and inhibits IFN-γ secretion from human T cells in a dose-dependent manner

**Fig. 1:**
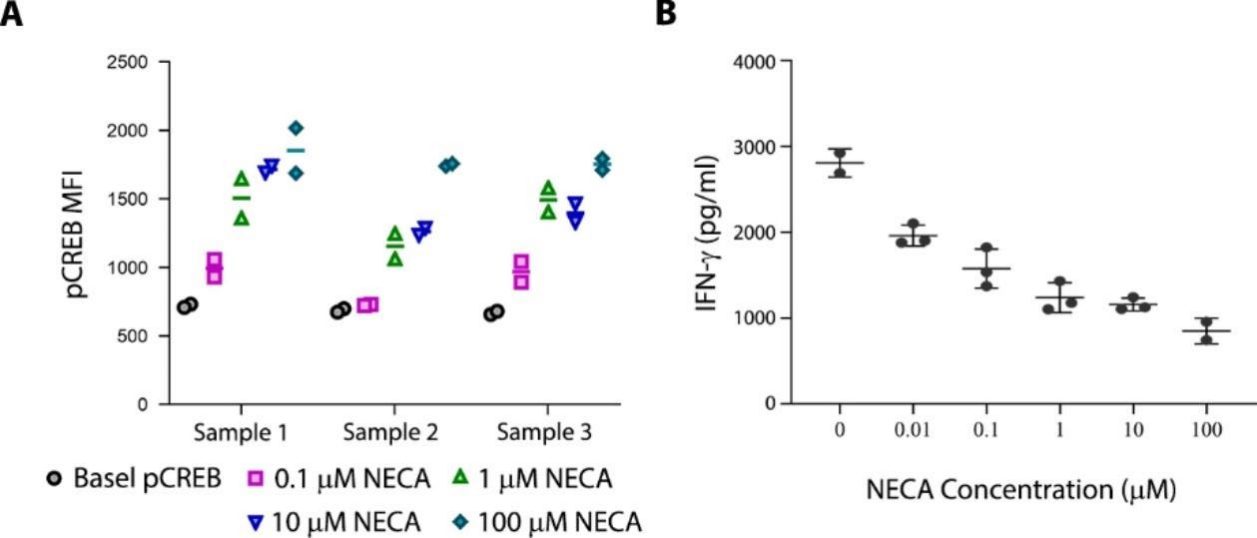
*Ex vivo* modulation of pCREB and IFN-γ in response to NECA. (A) NECA regulates CREB phosphorylation on CD3 + T cells in whole blood samples. Data from test samples from three different donors and plotted using GraphPad Prism. (B) NECA affects IFN-γ secretion in human PBMCs activated by anti-CD3/CD28 antibodies. The experiment was repeated at least 3 times, and the data shown here are from one representative experiment

Binding of adenosine to A2aR activates PKA, which directly phosphorylates transcriptional factor CREB. Thus, pCREB level reflects the adenosine-mediated pathway activation. We first sought to establish the quantitative relationship between adenosine concentration and its biological effect. As adenosine is not stable in human blood, with a biological half-life less than 10 s [34, 35], its stable analogue 5′-N-ethylcarboxamide adenosine (NECA) was used instead [36, 37]. Briefly, human whole blood samples from 3 donors were collected and mixed with different concentrations of NECA. The effect of NECA on phosphorylation of CREB in CD3 + T cells was measured by flow cytometry. IFN-γ secretion in anti-CD3/CD28 antibodies activated human PBMCs was measured by ELISA, with or without NECA treatment. Up to 100 µM tested, NECA stimulated CREB phosphorylation in a concentration dependent manner (Fig. 1 A). An inverse linear relationship was observed between cytokine secretion and NECA concentration (Fig. 1B). Taking together, these data underscored the significance of adenosine quantitative level at TME in modulating immune responses.

### DZD2269 is a potent and selective A2aR antagonist, which can completely abrogate adenosine-mediated immunosuppression even at high levels of adenosine

**Fig. 2:**
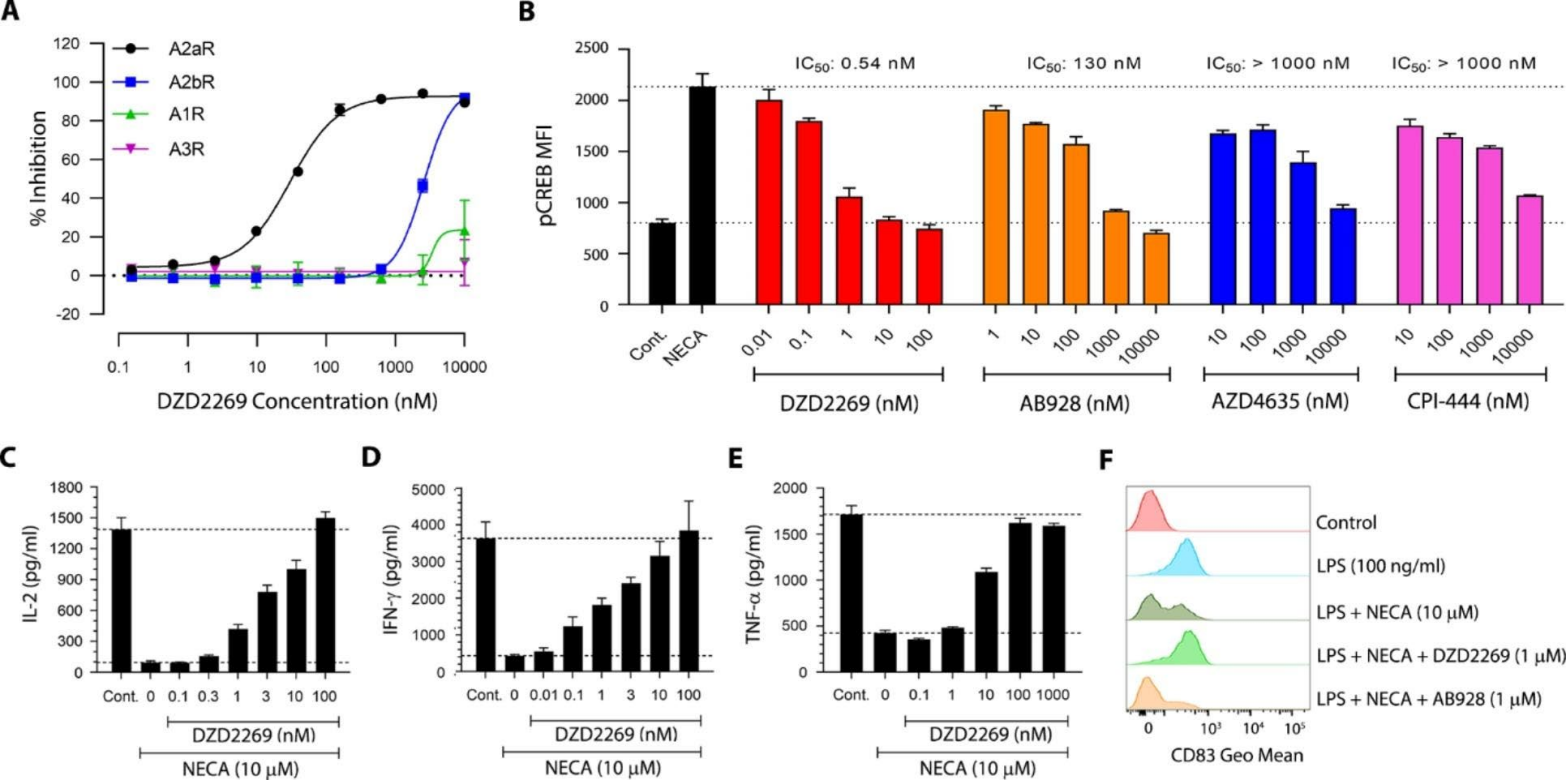
*In vitro* selectivity of DZD2269 against adenosine receptors and its role in relieving NECA-induced immunosuppression. (A) Effects of DZD2269 on 10 µM NECA-induced cAMP accumulation in CHO-A2aR and CHO-A2bR cells and calcium increase in CHO-A1R and CHO-A3R cells. (B) The ability of A2aR antagonists to block CREB phosphorylation in CD4 + T cells was measured by flow cytometry after stimulation of whole blood with 10 µM NECA. (C-E) DZD2269 relieved the inhibition of 10 µM NECA on the secretion of IL-2 (C), IFN-γ (D) and TNF-α (E) by activated PBMCs. (F) DZD2269 relieved the inhibition of DC maturation by 10 µM NECA. Each data point represents the mean of at least two technical replicates, and error bars represent SD. Each experiment was repeated at least three times, and the data shown here are from one representative experiment

The selectivity of DZD2269 on the four adenosine receptors, A2aR, A2bR, A1R and A3R, was assessed by intracellular cAMP production (A2aR and A2bR) and calcium mobilization assays (A1R and A3R) in CHO cells engineered to express individual adenosine receptors and G proteins. Both A2aR and A2bR are GPCRs coupled with G_s_ proteins. Binding of adenosine to both receptors activate AC through G_s_ proteins and increases intracellular cAMP levels. A1R and A3R signal through G_i/o_ to inhibit AC and decrease intracellular cAMP levels. In addition, A1R and A3R can also activate phospholipase C (PLC) and lead to increase of intracellular Ca^2+^ levels [18, 38, 39]. DZD2269 was over 100-fold more potent against A2aR vs. A2bR. Meanwhile, DZD2269 had little activities on A1R and A3R (Fig. 2 A and Table 1). In the same testing platform, the potency of DZD2269 was compared with other clinical A2aR antagonists (CPI-444, AZD4635 and AB928). At the lower concentrations of adenosine (1 µM adenosine or 0.1 µM NECA), all these compounds blocked cAMP production with IC_50_ around or below 50 nM. But at higher levels of adenosine (100 µM adenosine or 10 µM NECA), the levels reported in TME, their potencies were significantly reduced, making it impossible to cover the target clinically. Although the potency of DZD2269 also dropped with the increase of adenosine concentrations, it remained as the most potent A2aR inhibitor. Even at 100 µM adenosine, the EC_50_ of DZD2269 was 24 nM (Table 2), the drug exposure which is achievable in the clinic.

**Table 1:** In vitro activity of DZD2269 on four adenosine receptors

		Adenosine		NECA	
	Conc. (µM)	10	100	1	10
IC50 (nM)	A2aR	0.96 ± 0.36 (n = 23)	24 ± 4.6 (n = 6)	2.1 ± 0.39 (n = 6)	28 ± 4.2 (n = 6)
	A2bR	106 ± 16 (n = 8)	2042 ± 267 (n = 6)	217 ± 47 (n = 6)	2276 ± 388 (n = 6)
	A1R	8431 ± 1100 (n = 23)	> 10,000 (n = 6)	3235 ± 741 (n = 6)	> 10,000 (n = 6)
	A3R	> 10,000 (n = 8)	> 10,000 (n = 6)	> 10,000 (n = 6)	> 10,000 (n = 6)

Cells were stimulated with different concentrations of Adenosine or 5’-N-ethylcarboxamide adenosine (NECA). cAMP was used as a readout for CHO-A2aR and CHO-A2bR, intracellular calcium was used as a readout for CHO-A1R and CHO-A3R cells. Values are expressed as average ± SD of independent experiments

**Table 2:** In vitro inhibitory effect of A2aR antagonists on intracellular cAMP accumulation stimulated by adenosine or NECA

		Adenosine		NECA	
	Conc. (µM)	1	100	0.1	10
IC50 (nM)	DZD2269	0.10 ± 0.01 (n = 6)	24 ± 4.6 (n = 6)	0.14 ± 0.05 (n = 6)	28 ± 4.2 (n = 6)
	CPI-444	15 ± 7.0 (n = 6)	5831 ± 1881 (n = 6)	23 ± 3.8 (n = 6)	5653 ± 702 (n = 6)
	AZD4635	29 ± 11 (n = 4)	6314 ± 1691 (n = 4)	40 ± 5.9 (n = 4)	6534 ± 740 (n = 3)
	AB928	3.4 ± 0.92 (n = 3)	463 ± 90 (n = 3)	4.1 ± 1.2 (n = 3)	408 ± 25 (n = 3)

CHO-A2aR cells were stimulated with different concentrations of adenosine or NECA. cAMP was used as a readout. Values are expressed as average ± SD of independent experiments

In T cells isolated from human whole blood, the ability of DZD2269 to inhibit CREB phosphorylation in response to NECA stimulation was evaluated and compared with other A2aR antagonists. Treatment with A2aR antagonists (DZD2269, CPI-444, AZD4635 and AB928) led to a dose-dependent inhibition of CREB phosphorylation in both CD4+ (Fig. 2B) and CD8+ (Supplemental Fig. S3) T cells following stimulation of human whole blood with 10 µM NECA. The concentrations to achieve > 90% pCREB inhibition varied significantly among the antagonists. At 10 nM, DZD2269 completely abolished adenosine’s effect and brought pCREB to the control level. To achieve the same level of inhibition, over 1,000 nM AB928 was needed. In contrast, even at the highest concentration tested (10 µM), CPI-444 and AZD4635 failed to bring pCREB to the baseline (control) level.

The effect of DZD2269 to abrogate the immunosuppressive effects under high levels of NECA were assessed by measuring IL-2, IFN-γ, and TNF-α production from activated human PBMCs. NECA at 10 µM completely blocked IL-2, IFN-γ and TNF-α production, while DZD2269 abrogated the suppression in a concentration-dependent manner (Fig. 2, C to E). About 100 nM DZD2269 was needed to fully restore the cytokine production.

Monocytes from human PBMCs were isolated and stimulated with GM-CSF and IL-4 to produce immature DCs (iDCs), which were then treated with LPS to promote their maturation. As showed in Fig. 2 F, LPS stimulation converted all iDCs to mature DCs, as measured by CD83, a surface marker that distinguishes mature DCs from iDCs [40, 41]. NECA suppressed the stimulatory effect by LPS and lowered the proportion of mature DCs. DZD2269 almost completely abolished NECA’s suppression. Interestingly, although both A2aR and A2bR have been reported to be involved in the inhibitory effect of adenosine on DC maturation, the A2aR/A2bR dual antagonist AB928 did not relieve the inhibitory effect by NECA [42], suggesting this effect was mainly driven by A2aR.

### T cells are required for anti-tumor effect of DZD2269

**Fig. 3:**
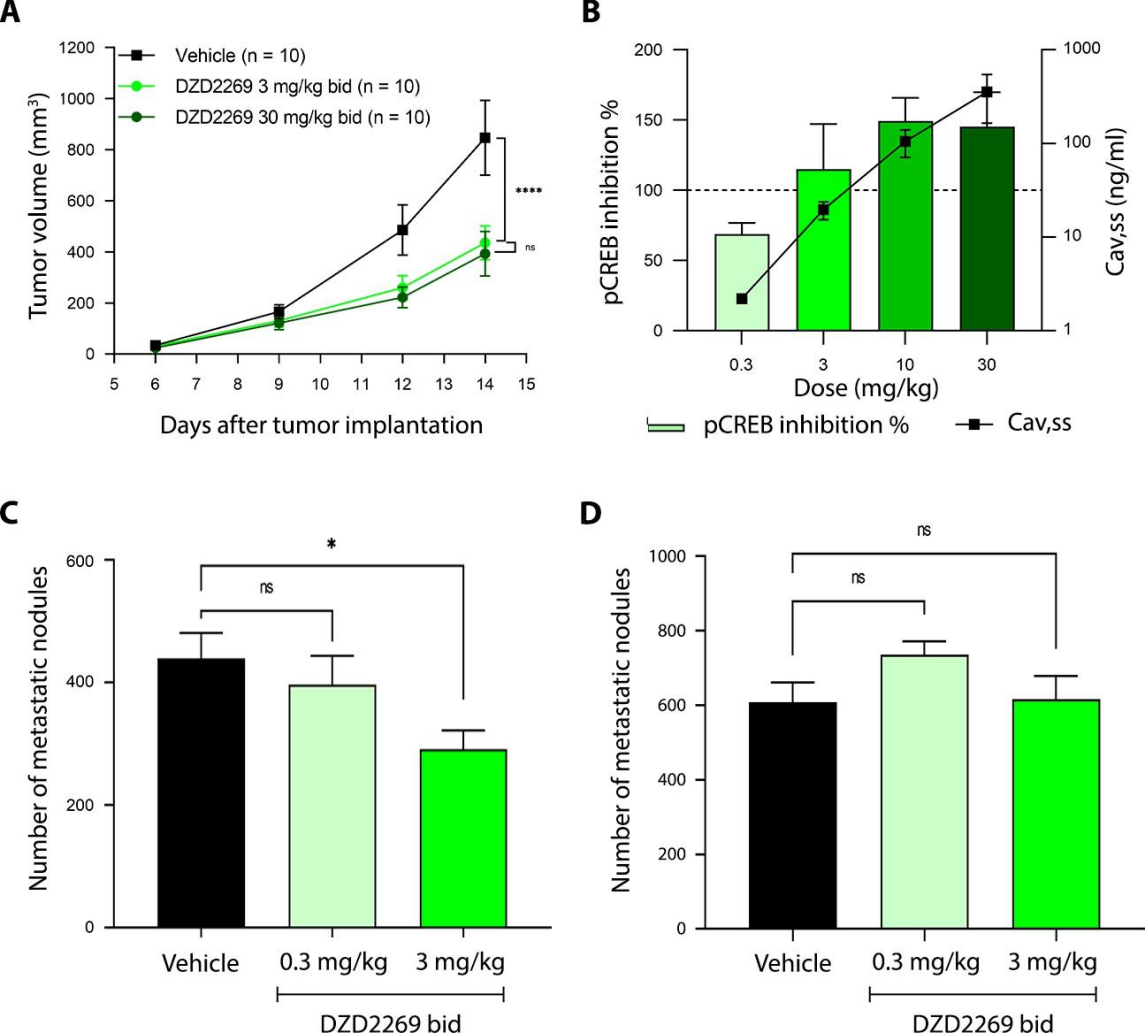
Anti-tumor efficacy of DZD2269 in animal models in the presence or absence of T cells (A) B16F10 cells were inoculated in the back of C57BL/6 mice, and the mice were treated with DZD2269. Statistically significant difference was calculated by using two-way ANOVA (**** P: < 0.0001, ns: no significance). (B) C57BL/6 mice were treated with DZD2269 at 0.3, 3, 10, and 30 mg/kg bid for 4 days. On day 4, plasma was collected for PK analysis before dosing and at 0.5, 1, 2, 4, and 7 h after dosing. On the same day, whole blood was also collected before dosing for PD (pCREB) analysis. Cav, ss: average steady-state plasma drug concentration. (C-D) B16F10 cells were injected through tail vein on (C) C57BL/6 mice (n = 9) or (D) BALB/c nude mice (n = 8), lung metastatic nodes were counted at Day13. bid: twice daily. Statistically significant difference was calculated by using one-way ANOVA (*: p < 0.05; ns: no significance)

The anti-tumor efficacy of DZD2269 was evaluated in a syngeneic mouse tumor model. As monotherapy, DZD2269 significantly inhibited tumor growth at the dose of 3 mg/kg. Further increasing dose to 30 mg/kg only marginally enhanced its efficacy (Fig. 3 A). Consistent with the efficacy results, steady-state plasma drug concentration at 3 mg/kg was sufficient to achieve over 90% inhibition of pCREB in T cells (Fig. 3B). In addition to inhibiting tumor growth, DZD2269 showed potent anti-metastasis effect in immunocompetent C57BL/6 mice, as demonstrated in B16F10 melanoma lung metastasis model. At 3 mg/kg, DZD2269 significantly decreased the number of tumor nodules metastasized to the lung (Fig. 3 C). When the same experiment was carried out in BALB/c nude mice lacking thymus and unable to produce T cells, DZD2269 lost its anti-metastasis effect totally (Fig. 3D), suggesting functional T cells were required for its anti-metastasis effect.

### DZD2269 enhances anti-tumor effects in combination with radiotherapy

**Fig. 4:**
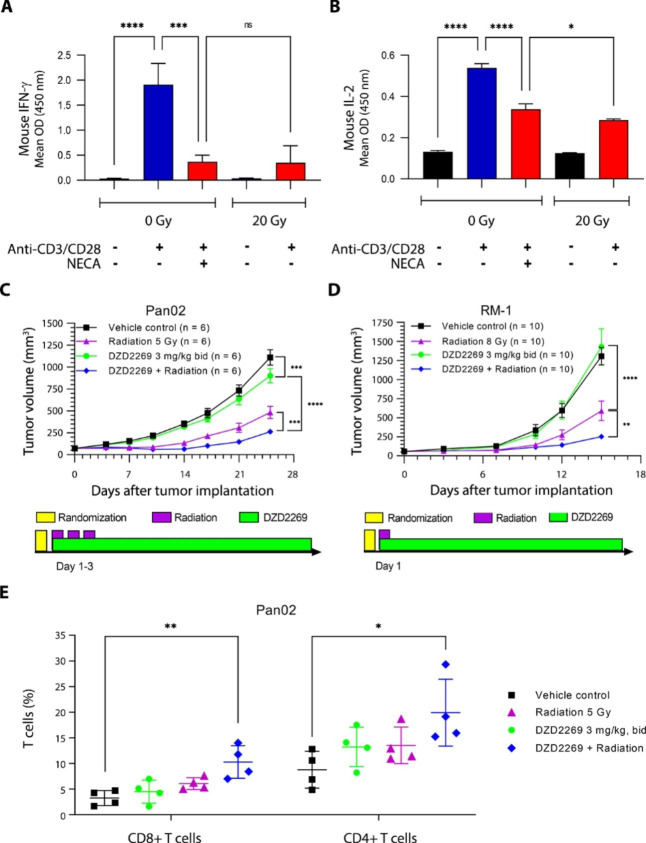
DZD2269 enhances the efficacy of radiotherapy (RT) in syngeneic mouse in vivo models. (A-B) RM-1 tumor cells were irradiated with 20 Gy radiation and were co-cultured anti-CD3/CD28 antibodies activated mouse splenocytes. (A) IFN-γ in supernatant was measured 72 h after co-culture. (B) IL-2 in supernatant was measured 24 h after co-culture. Significance Statistically significant difference was calculated by using one way ANOVA (*: p < 0.05, ***: p < 0.001, ****: p < 0.0001, ns: no significance). (C) Pancreatic Pan02 and (D) Prostate RM-1 models were established in C57BL/6 mice. Pan02 tumor-bearing mice received daily fixed 5 Gy radiation at day 1, day 2 and day 3 and RM-1 tumor-bearing mice received 8 Gy radiation at day 1. DZD2269 at the indicated dosage was given from day 1 to the end of the experiment. Error bars represent standard error of the mean. Statistically significant difference was calculated by using two-way ANOVA (*: p < 0.05, **: p < 0.01, ***: p < 0.001, ****: p < 0.0001). bid: twice daily. (F) Tumor samples from Pan02 model were harvested at the end of the experiment. Infiltrated CD8 + and CD4 + T cells were determined by IHC. N = 4 mice per group. One way ANOVA was conducted to compare the combination group versus vehicle or single-agent groups (*: p < 0.05, **: p < 0.01)

Radiation can lead to massive cell death quickly, consequently elevating adenosine concentration at TME and inducing immunosuppression. It is thus hypothesized that the combination of DZD2269 and radiation should have synergistic effect. Mouse prostate cancer cells (RM-1) were irradiated (20 Gy) and co-cultured with mouse splenocytes stimulated with anti-CD3/CD28 antibodies. IL-2 and IFN-γ in the supernatant were measured 24 and 72 h after co-culture, respectively. Naïve mouse splenocytes produced minimal amount of IFN-γ (Fig. 4 A) and IL-2 (Fig. 4B) when co-cultured with unirradiated tumor cells. Anti-CD3/CD28 antibody stimulation significantly induced the production of these cytokines, and the production of these cytokines was inhibited by the addition of NECA. Whereas, when irradiated tumor cells were mixed with the activated mouse splenocytes, production of IL-2 and IFN-γ was blocked. Notably, the extent of inhibition with 20 Gy radiation was similar to that of 10 µM NECA.

The combination effect of radiotherapy and DZD2269 was further tested in several subcutaneous mouse models: RM-1 (prostate cancer) and Pan02 (pancreatic cancer). Due to the different intrinsic sensitivities to radiation in these animal models, the dose and frequency of radiation were adjusted to produce about 50% tumor growth inhibition, leaving rooms for observing synergies between radiation and DZD2269. Approximately 20% tumor growth inhibition was observed with DZD2269 monotherapy in Pan02 model (Fig. 4 C), but little effect was observed in RM-1 model (Fig. 4D). When radiation and DZD2269 were given simultaneously, a significant synergistic effect was observed. In addition, there was no obvious body weight loss with the combination treatment (Data not shown).

In Pan02 model, few CD8 + and CD4 + T cells were present in the TME. Tumor remained “cold” after DZD2269 treatment. Radiation increased the number of both CD8 + and CD4 + T cells in TME, while most significant increase of infiltrated T cells was found only in radiation and DZD2269 combination group (Fig. 4E).

### DZD2269 in combination with anti-PD-1 or oxaliplatin showed synergistic anti-tumor effect *in vivo*

**Fig. 5:**
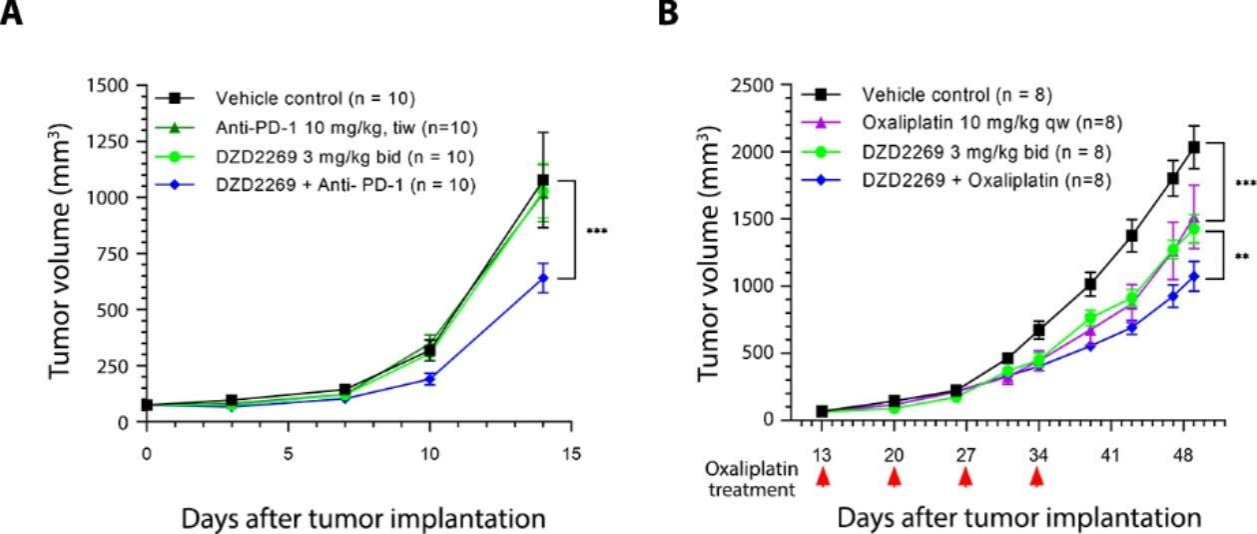
DZD2269 synergizes with anti-PD-1 antibody or oxaliplatin in syngeneic mouse in vivo model. (A) Prostate RM-1 tumor-bearing mice were treated with 10 mg/kg anti-PD-1 antibody, with or without DZD2269 (3 mg/kg). (B) Pancreatic Pan02 tumor-bearing mice were treated with 10 mg/kg oxaliplatin with or without DZD2269 (3 mg/kg). bid: twice daily, qw: once a week. tiw: three times a week. Statistically significant difference was calculated by using two-way ANOVA (**: p < 0.01, ***: p < 0.001)

Anti-tumor efficacy of DZD2269 in combination with anti-PD-1 antibody was evaluated in syngeneic prostate RM-1 mouse model. DZD2269 (3 mg/kg) alone or anti-PD-1 antibody (10 mg/kg) alone did not inhibit the growth of RM-1 tumors, but administration of DZD2269 and anti-PD-1 antibody together significantly enhanced the anti-tumor effect (Fig. 5 A).

Similar synergistic effect with chemotherapy oxaliplatin was observed. As shown in Fig. 5B, oxaliplatin or DZD2269 alone showed some anti-tumor effect, while in combination, the anti-tumor efficacy was significantly enhanced.

### Data from an ongoing phase 1 study showed DZD2269 as a potent A2aR inhibitor with positive exposure-response relationship

**Fig. 6:**
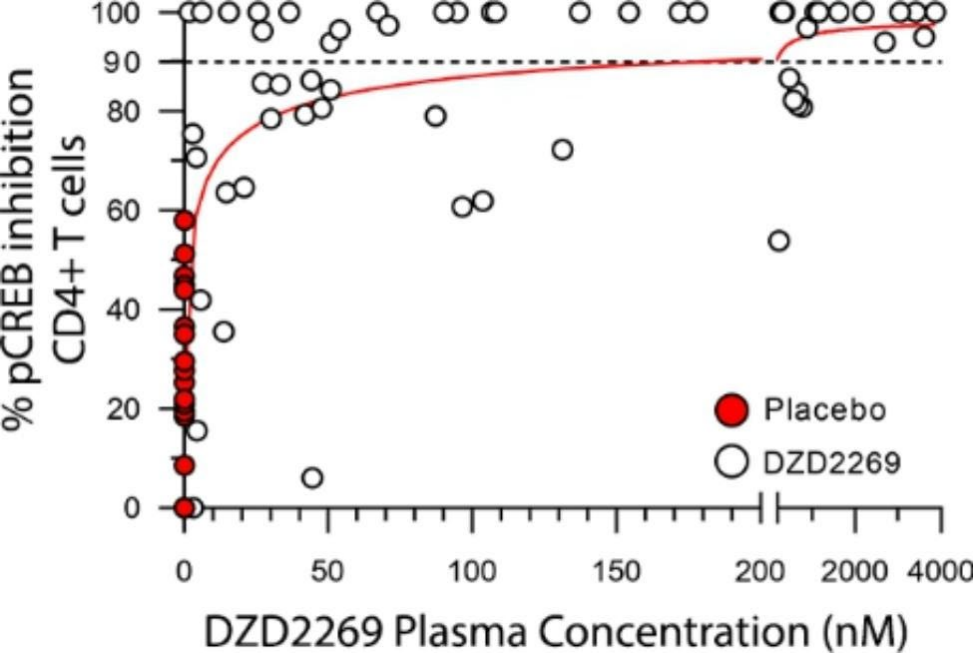
Correlation of DZD2269 plasma concentration (PK) and pCREB inhibition (PD) on CD4 + T Cell. Whole blood samples were collected at pre-dose, 2 h, and 24 h after single dose of DZD2269, samples were stimulated with or without 10 µM NECA. pCREB inhibition was calculated for each PD time point and plotted against PK at the corresponding time point. pCREB data from placebo-treated subjects (red dot) were also included in the analysis to enable assessment of biological and technical assay variation in PD assay. The dashed black line indicates the 90% pCREB inhibition

A phase 1 clinical study is being conducted in healthy volunteers to evaluate safety, PK and PD of DZD2269 (NCT04932005). To determine the effect of DZD2269 on blocking the A2aR pathway in T cells, pCREB was measured in T cells in whole blood samples collected at different time points post dosing, with or without ex vivo stimulation by 10 µM NECA. The pCREB levels of the post-dose samples were compared to the pCREB levels of the pre-dose samples to calculate the percentage of inhibition. pCREB inhibition was then plotted against plasma drug concentration to evaluate the PK/PD relationship. As the data showed, a clear positive PK/PD relationship was demonstrated in this phase 1 study: the higher the plasma concentration of DZD2269, the stronger the inhibition of pCREB. The same trend was observed in both CD4+ (Fig. 6) and CD8 + T cells (Supplemental Fig. S4). About 90% inhibition of pCREB was reached when DZD2269 total plasma concentration is around 180 nM. Meanwhile, no drug related adverse events were reported up to 160 mg (with plasma C_max_ concentration about 4 µM), the highest dose tested.

## Discussion

As a potent immune suppressor, extracellular adenosine concentration is low under the normal physiological conditions, in the range of 10–100 nM [43]. In response to hypoxia, inflammation, and cell death, its level can rapidly increase over one thousand folds [3, 43]. Our data showed (Fig. 1, A and B) that NECA concentration of 0.1 µM to 100 µM (equivalent to 1 µM to 1 mM adenosine) resulted in upregulation of pCREB and downregulation of IFN-γ in a linearly fashion. Even at the highest concentration tested, the effects on pCREB and IFN-γ signals were still not saturated, suggesting the system has well evolved to quantitatively respond to adenosine concentration change over a wide range. An ideal A2aR inhibitor should therefore be able to fully block adenosine-mediated immunosuppression over a range of adenosine concentrations. Current small molecule A2aR inhibitors exert their effect through competing against adenosine binding to A2aR. One major limitation of these first generation A2aR inhibitors is that their ability to block A2aR dropping rapidly with the increase of adenosine concentrations. At the therapeutically relevant adenosine level found in TME, their potency becomes so weak that it is impossible to achieve the level of exposure needed to block the pathway (Table 1). This could be one of the reasons why these A2aR inhibitors showed some efficacy in clinical trials, but not as significant as expected based on the known biology.

DZD2269 was designed as an A2aR antagonist able to overcome high level of adenosine mediated immunosuppression. At the adenosine level found in TME relevant (100 µM adenosine or 10 µM NECA), DZD2269 could still effectively block A2aR, while other inhibitors lost most of their activities (Fig. 2B). This unique property, in addition to its excellent clinical safety profile, provides an opportunity to explore the full effect of blocking adenosine/A2aR pathway in the clinic. In the ongoing phase 1 study, the plasma concentration of DZD2269 at about 180 nM correlated with over 90% inhibition of pCREB. Even at the highest exposure of 4 µM in the plasma, no drug related adverse events were reported, confirming its excellent safety profile.

Based on known mechanism, A2aR antagonists exert their function by antagonizing adenosine’s immune repression. A robust host anti-tumor immunity is therefore needed to realize the benefit. Clinical trial results released from the current adenosine pathway inhibitors are mainly from studies in patients with advanced cancer who have failed multiple lines of therapy. Patients with early disease and more robust immune functions should benefit more from adenosine pathway blockers. In this aspect, it is especially interesting to find the synergistic effect of DZD2269 with radiation. Patients eligible for radiation treatment in practice tend to have early and local diseases and are best suited for validating adenosine pathway inhibitors. Ionizing radiation is one of the most efficient ways of killing cells and expected to increase extracellular adenosine rapidly to a high level. Consistently, treatment with 20 Gy radiation and 10 µM NECA showed similar level of suppression on IFN-γ and IL-2 production by T cells (Fig. 4, A and B). Thus, being able to overcome high level of adenosine-mediated suppression is especially critical to achieve the synergistic benefit in this setting. To our knowledge, DZD2269 is the only adenosine pathway inhibitor demonstrating synergistic anti-tumor effect with radiation.

Ionizing radiation can cause tumor cell death through apoptosis, necrosis, autophagy, and mitotic catastrophe [44, 45], which can lead to the release of pro-inflammatory cytokines, chemokines, and tumor neoantigens, thereby enhancing the immunogenicity of tumors. The released pro-inflammatory mediators, also known as damage-associated molecular patterns (DAMPs), are recognized by DCs and influence DC differentiation and maturation [46]. iDCs possess strong antigen capture ability but lack the co-stimulatory molecules involved in antigen presentation and lymphocyte activation. They need to be “matured” to be able to effectively stimulate the proliferation and differentiation of T cells. Adenosine or NECA can inhibit DCs’ maturation while DZD2269 can effectively abrogate the inhibitory effect (Fig. 2E). Our data provided further evidence that DCs and innate immune cells also contribute to the synergistic anti-tumor effect observed with radiation in vivo.

Anti-PD-1 treatment significantly increases A2aR expression in T cells [22]. In general, response to immune checkpoint inhibitors (ICIs) requires functional IFN-γ responsive genes, and defects in IFN-γ signature genes have been reported in patients resistant to ICIs [47]. Adenosine/A2aR-mediated signaling in effector T cells markedly decrease IFN-γ production. In all models we tested, DZD2269 leads to robust IFN-γ induction. These data point to adenosine signaling as a nexus of cancer immune responses. Consistently, DZD2269 in combination with anti-PD-1 antibody showed synergistic anti-tumor effect in a model resistant to either anti-PD-1 treatment or DZD2269 alone (Fig. 5 A). This exciting potential is being tested in the clinic.

## Conclusion

In conclusion, our work demonstrates that DZD2269 is a potent and selective A2aR antagonist. Whether used as a single agent or in combination with radiation, chemotherapy, or immunotherapy, DZD2269 can overcome a wide range of adenosine-induced immunosuppression and enhance the anti-tumor efficacy of these treatments. DZD2269 showed good PK/PD correlation and safety profile in an ongoing phase 1 study. These data support further clinical development of DZD2269 in patients with cancer.

## Electronic supplementary material

Below is the link to the electronic supplementary material.

Supplementary Material 1

## Data Availability

The datasets used and/or analyzed during the current study are available from the corresponding author on reasonable request.

## Declarations

### Author details

^1^Biomed-X Center, Academy for Advanced Interdisciplinary Studies, Peking University, Beijing, 100871, China. ^2^Dizal Pharmaceuticals, Shanghai, 201203, China.

## Abbreviations

AC: Adenylyl cyclase
ALP: Alkaline phosphatase
cAMP: cyclic adenosine monophosphate
CREB: cyclic AMP response element binding protein
DCs: Dendritic cells
ICIs: Immune checkpoint inhibitors
iDCs: Immature DCs
PAP: Prostatic acid phosphatase
MDSCs: Myeloid-derived suppressor cells
NECA: 5’-N-ethylcarboxamide adenosine
NKT: Natural killer T cell
PBMCs: Peripheral blood mononuclear cells
PD: Pharmacodynamics
PLC: Phospholipase C
PK: Pharmacokinetics
PKA: cAMP-dependent protein kinase A
ROS: Reactive oxygen species
TME: Tumor microenvironment
Treg: Regulatory T cells
