# Supplementary material for: Overcoming high level adenosine-mediated immunosuppression by DZD2269, a potent and selective A2aR antagonist
Source: J Exp Clin Cancer Res. 2022 Oct 14;41:302. doi: 10.1186/s13046-022-02511-1 (PMC9563815; doi:10.1186/s13046-022-02511-1)
Supplement: Supplementary file 1 — Supplementary Material 1 [file 13046_2022_2511_MOESM1_ESM.pdf]

Supplementary Materials

Figure S1.

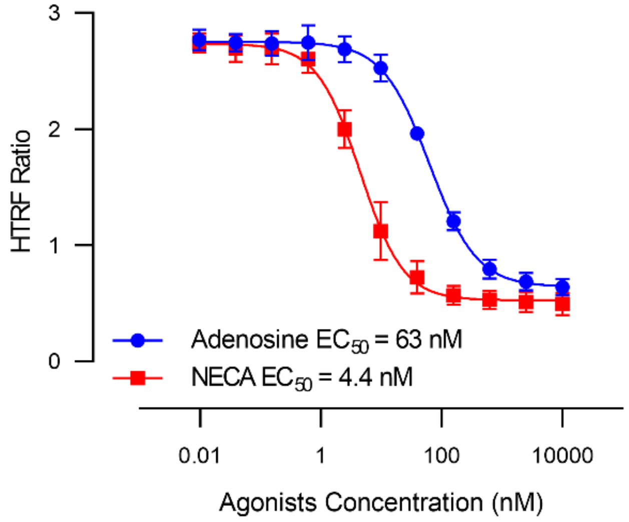

Fig. S1. cAMP accumulation in response to adenosine and NECA. CHO-A2aR cells were treated with various concentration of adenosine or NECA to stimulate cAMP production. Native cAMP produced by cells compete with d2-labeled cAMP for binding to monoclonal cAMP Europium Cryptate labeled antibody (Europium donor). The specific signal is inversely proportional to the concentration of cAMP in the samples.

Figure S2.

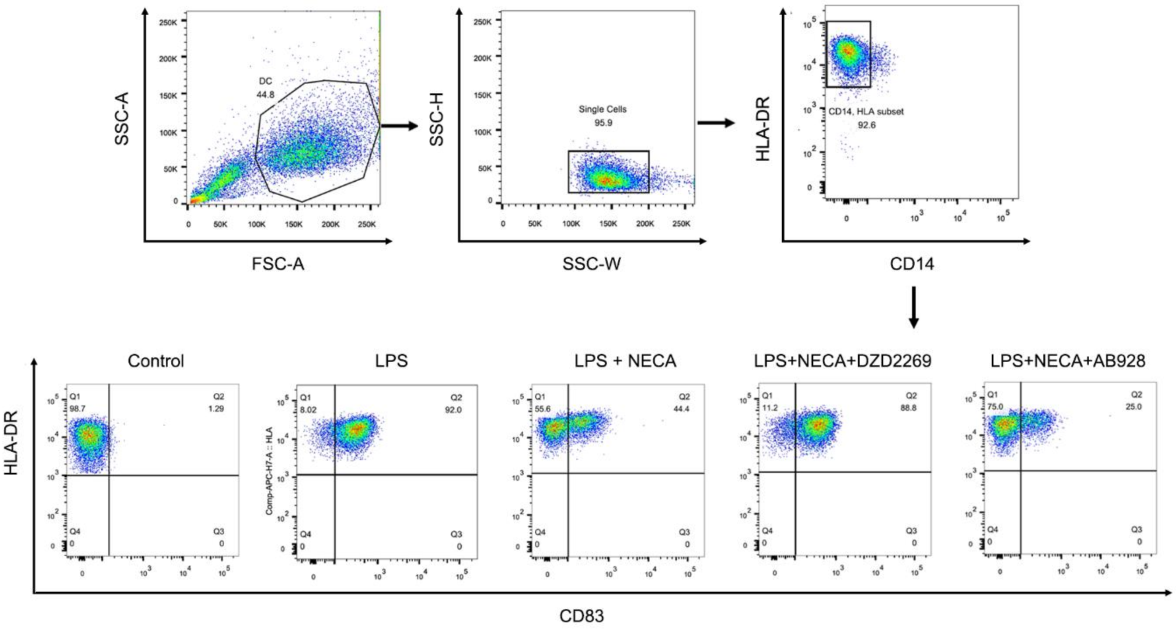

Fig. S2. Gating strategy of matured DC after LPS stimulation. Human $CD14^{lo}$ and $HLA\text{-}DR^{hi}$ DC were gated from LPS-stimulated DCs, matured DC was measured by CD83 expression level.

Figure S3.

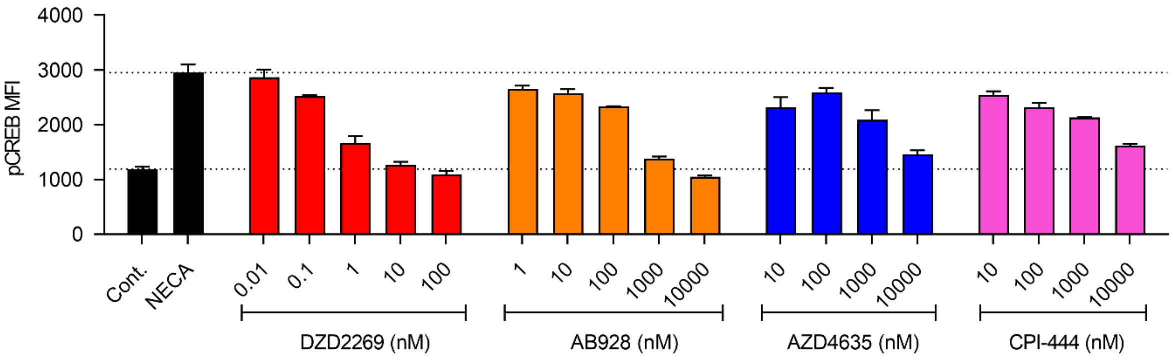

Fig. S3. A2aR antagonist blocks CREB phosphorylation in CD8+ T cells. Whole blood samples were incubated with A2aR antagonists for 30 minutes, then stimulated with 10 µM NECA for 15 minutes, and CREB phosphorylation in CD8+ T cells was measured by flow cytometry after stimulation.

Figure S4.

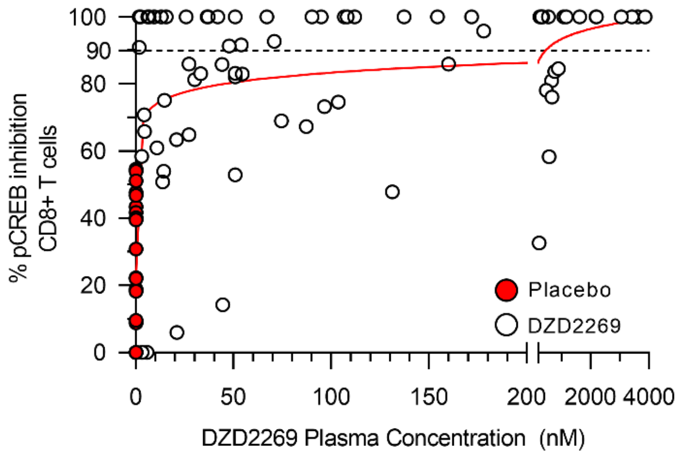

Fig. S4. Correlation of DZD2269 plasma concentration (PK) and pCREB inhibition (PD) on CD8+ T Cell. Whole blood samples were collected at pre-dose, 2 hours and 24 hours after single dose of DZD2269, samples were stimulated with or without 10 µM NECA. pCREB inhibition was calculated for each PD time point and plotted against PK at the corresponding time point. pCREB data from placebo-treated subjects (red dot) were also included in the analysis to enable assessment of biological and technical assay variation in PD assay. The dashed black line indicates the 90% pCREB inhibition.
